# Content of polycyclic aromatic hydrocarbons in soil in a multi-annual fertilisation regime

**DOI:** 10.1007/s10661-020-08252-y

**Published:** 2020-04-27

**Authors:** Ewa Mackiewicz-Walec, Sławomir Józef Krzebietke

**Affiliations:** Department of Agricultural Chemistry and Environmental Protection, Faculty of Environmental Management and Agriculture, University of Warmia and Mazury in Olsztyn, 10-719 Olsztyn, Poland

**Keywords:** PAHs, Manure mineral fertilisation, Soil multi-annual experiment

## Abstract

The study assessed changes in the total 16 PAH (polycyclic aromatic hydrocarbon) content of soil which occurred in 1998–2009, during a multi-annual, manure-mineral and mineral fertilisation experiment, carried out in Bałcyny near Ostróda (Poland), according to a design unchanged since 1986 The study focused on the impact of multi-annual, diversified mineral fertilisation (N, P, K, Mg and Ca) compared to manure applied every two years at a dose of 40 t/ha. The four plants used in the crop rotation included sugar beet, spring barley, maize and spring wheat. The content of the total 16 polycyclic aromatic hydrocarbons was significantly higher in the manure-fertilised soil than in the soil fertilised with mineral fertilisers only. Under the regular manure fertilisation conditions, liming of the soil significantly increased the total 16 PAH content, and the lowest dose of nitrogen significantly decreased its PAH content. The lowest nitrogen dose had an opposite effect in the soil fertilised with mineral fertilisers only, where it caused a significant increase in the content of the PAHs. However, the increased doses of nitrogen also resulted in an increase in the PAH content in the soil fertilised with manure and without this fertiliser.

## Introduction

In view of global environmental pollution, it is assumed that toxic compounds originating from natural sources (the so-called natural background) account for a small proportion of all pollutants, nowadays mainly generated by human activities. Contaminants include polycyclic aromatic hydrocarbons (PAHs), chlorophenols, polychlorinated biphenyls (PCB), dioxins (PCDD, PCDF) and certain pesticides. The hazard to humans posed by these substances is primarily due to their capability of penetrating into the food chain following the consumption of vegetable and animal products originating from contaminated (urbanised) areas as well as crops from plantations fertilised with sewage sludge and various organic fertilisers. Some of these compounds have a strong carcinogenic and mutagenic effect and pose a serious threat to human health and life. In practice, there are natural sources of certain compounds (e.g. PAHs) which may be present in the soil as a result of incomplete organic matter mineralisation. Various patterns of land development or soil use may result in significant changes in the PAH content.

Polycyclic aromatic hydrocarbons are of low lability; they are difficult to degrade physically or chemically, and only microorganisms and soil enzymes are capable of decomposing their benzene ring chains (Miles and Doucette 2001; Klimkowicz-Pawlas and Maliszewska-Kordybach 2003; Ogbonna et al. 2013; Borowik and Wyszkowska 2018). The development of soil microflora capable of decomposing these undesirable compounds is primarily determined by the availability of nutrients, particularly not only nitrogen but also P, K, S, Ca and Mg (Maliszewska-Kordybach et al. 2007; Amezcua-Allieri et al. 2012).

Organic matter reduces the bioavailability of PAHs and thus mitigates their adverse effects (Ockenden et al. 2003; Doick et al. 2005; Ouvrard et al. 2014). This is the effect of the adsorption of PAHs on organic molecules, leading to limited oxidation and bioavailability for microorganisms (Maliszewska-Kordybach 1991). According to Ogbonna et al. (2013), both manure and NPK fertilisation effectively biodegraded most PAH group compounds (over a period of 21 days) except benzo[a]pyrene and indeno[1,2,3-cd]pyrene. Manure, however, exerted a stronger biodegradation effect than mineral (NPK) fertilisers.

The majority of studies concerns the high content of PAHs from pyrogenic and petrogenic sources, i.e. associated with combustion of petroleum, wood or coal (e.g. Kaushik et al. 2012; Downard et al. 2015) and with substances derived from crude oil (e.g. Nganje et al. 2007; Abdel-Shafy and Mansour 2016), respectively. The PAH sources also include biogenic natural processes such as decomposition of organic matter in oil seeps (Pampanin and Sydnes 2013), wood of tropical forests (Krauss et al. 2005) or decay of pine needles (Ratola et al. 2006). The research concerning the long-term effects of natural fertilisers commonly used in plant fertilisation and their interactions with mineral fertilisation is still very scarce.

The aim of the study was to assess the impact of 12-year (manure and mineral) fertilisation in a multi-annual static experiment carried out in an unchanged design on the total 16 PAH content in the 0–30 cm soil layer. We hypothesised that long-term mineral fertilisation (1) with manure will increase the content of the total 16 PAHs in soil and (2) without manure will decrease the content of the total 16 PAHs in soil.

## Material and methods

### Description of the field experiment

A multi-annual controlled fertilisation experiment has been carried out in Bałcyny (N: 53° 35′ 38.1″, E: 19° 50′ 56.1″) near Ostróda (Poland) in an unchanged design for 33 years. The experimental field lies on a Haplic Luvisol soil. Based on the particle-size distribution, the soil was classified as sandy loam (Table 1). Before the experiment, the soil contained 100.0 mg K; 53.2 mg Mg; 41.3 mg P; 7.9 g of organic carbon and 0.79 g of total nitrogen in a kilogram of soil, and the reaction was slightly acid: pH KCl (1 mol/dm^3^) = 6.2. The study focused on the impact of multi-annual diversified mineral fertilisation (N, P, K, Mg and Ca) compared to manure applied every two years at a dose of 40 t/ha. The four plants used in the crop rotation compared included sugar beet, spring barley, maize and spring wheat. Manure was always used in maize and sugar beet cultivation. Liming was always carried out after the completion of a rotation cycle at a dose of 2500 kg CaO/ha. Phosphorus fertilisation (triple granuled superphosphate—46% P_2_O_5_) was applied depending on the crops under cultivation (sugar beet, spring wheat, spring barley—34.9 kg P/ha; maize—26.2 kg P/ha). The experiment comprised three nitrogen fertilisation system (ammonium nitrate—34% N) levels (N_1_ 60, N_2_ 120, N_3_ 180 kg N/ha—sugar beet, maize; N_1_ 30, N_2_ 60, N_3_ 90 kg N/ha—spring barley; N_1_ 40, N_2_ 80, N_3_ 120 kg N/ha—spring wheat) and potassium (potassium salt—60% K_2_O)—multiple in three doses (K_1_ 66.4 kg, K_2_ 132.8, K_3_ 199.2 kg K/ha—sugar beet; K_1_ 33.2, K_2_ 66.4, K_3_ 99.6 kg K/ha, spring barley; K_1_ 49.8, K_2_ 99.6, K_3_ 149.4 kg K/ha—maize and K_1_ 24.9, K_2_ 49.8, K_3_ 74.7 kg K/ha for spring wheat) (Table 2).

**Table 1:** Particle size distribution (Particle size (…) 2008. 2009)

Soil horizon	Sampling depth (cm)	Particle size (mm) distribution (%)									Texture class (USDA^a^)
		> 2	2–1	1–0.5	0.5–0.25	0.25–0.1	0.1–0.05	0.05–0.02	0.02–0.002	< 0.002	
Ap	5–15	3	2	3	10	34	17	8	20	6	Sandy loam (SL)
A2	35–45	3	3	5	10	32	15	8	22	5	
											Sandy loam (SL)
B	60–70	1	2	3	11	36	13	2	17	16	
											Sandy loam (SL)
C	115–125	4	2	4	11	40	7	2	16	18	Sandy loam (SL)

^a^United States Department of Agriculture (USDA)

**Table 2:** Mineral fertilisation regime

No	Variant	SUGAR BEET*Spring barley***Maize** Spring wheat*												
		N				P		K				Mg		
		Dose [kg/ha]												
1	N_0_P_0_K_0_	0	*0*	**0**	0*	0*	**0**	0	0	**0**	0*	0	0*	**0**
2	N_1_P_1_K_1_	60	*30*	**60**	40*	34.9*	**26.2**	66.4	*33.2*	**49.8**	24.9*	0	0*	**0**
3	N_2_P_1_K_1_	120	*60*	**120**	80*	34.9*	**26.2**	66.4	*33.2*	**49.8**	24.9*	0	0*	**0**
4	N_3_P_1_K_1_	180	*90*	**180**	120*	34.9*	**26.2**	66.4	*33.2*	**49.8**	24.9*	0	0*	**0**
5	N_2_P_1_K_2_	120	*60*	**120**	80*	34.9*	**26.2**	132.8	*66.4*	**99.7**	49.8*	0	0*	**0**
6	N_2_P_1_K_3_	120	*60*	**120**	80*	34.9*	**26.2**	199.3	*99.7*	**149.7**	74.7*	0	0*	**0**
7	N_2_P_1_K_2_Mg	120	*60*	**120**	80*	34.9*	**26.2**	132.8	*66.4*	**99.7**	49.8*	48.2	18.1*	**24.1**
8	N_2_P1K_2_MgCa	120	*60*	**120**	80*	34.9*	**26.2**	132.8	*66.4*	**99.7**	49.8*	48.2	18.1*	**24.1**

### Analytical methods

In 1998–2009, soil samples were collected using a soil probe from each object after the growing period of the crop cultivated in a particular year. After drying, the soil was passed through a 2 mm mesh sieve.

The total 16 PAHs included naphthalene, acenaphthene, acenaphthylene, fluorene, phenanthrene, anthracene, fluoranthene, pyrene, benzo(a)anthracene, chrysene, benzo(b)fluoranthene, benzo(k)fluoranthene, benzo(a)pyrene, indeno(1,2,3-cd)pyrene, dibenzo(a,h)anthracene and benzo(g,h,i)perylene. Their content was determined using a Trace GC Ultra ITQ900 (THERMO) gas chromatograph with a FID detector. An analysis of the total 16 polycyclic aromatic hydrocarbon contents was carried out after 1-h extraction of 20 g of soil with 20 cm^3^ of acetonitrile using an ultrasonic cleaner and horizontal shaker. The extract (10 cm^3^) was decanted and purified using a MPW-350R centrifuge, a SPE solid-phase extraction unit and a SPE-NH_2_/C18 column with a 1500-mg bed and a capacity of 6 cm^3^. Methanol in an amount of 10 cm^3^ was used to rinse out PAHs from the bed; the extract was then concentrated to a volume of 0.2 cm^3^ in the presence of nitrogen. The assays were carried out on a 30-m-long Rxi-5ms column with an internal diameter of 0.25-mm ID. The walls of the column were coated with a carrier saturated with liquid stationary phase (SCOT) having the stationary layer 0.25 μm thick. The carrier gas was He passed at a constant flow rate of 3 cm^3^ min^−1^ as well as H_2_, air and N_2_, respectively (35, 350 and 30 cm^3^/min). The following temperature programme was applied: 0–100 °C—0.2 min; 50 °C min^−1^—143 °C—1.5 min; 8 °C min^−1^—180 °C—0.4 min; 100 °C min^−1^—210 °C—1.5 min; 10 °C min^−1^—300 °C—5 min = 23.39 min. The detector was set at 340 °C, while the splitless injector temperature was 250 °C. Calibration was carried out based on a standard solution manufactured by Restek Corporation, containing a mixture of the total 16 PAHs with a concentration of 2000 μg/cm each. The rate of PAH recovery from the soil ranged from 84 to 93%, considered separately for each compound.

### Mineral composition and the total 16 PAHs in manure

The mean content of basic elements in the dry matter of manure was as follows: phosphorus—6.7 g/kg; calcium—18.2 g/kg; magnesium—6.1 g/kg; nitrogen—17.5 g/kg; potassium—11.4 g/kg; sulphur—4.9 g/kg, cadmium (Cd)—0.26 mg/kg; copper (Cu)—36.8 mg/kg; nickel (Ni)—6.64 mg/kg; lead (Pb) < 2.50 mg/kg; zinc (Zn)—223 mg/kg; mercury (Hg)—0.063 mg/kg; cobalt (Co) < 5.00 mg/kg; manganese (Mn)—334 mg/kg; molybdenum (Mo) < 5.00 mg/kg; and chromium (Cr)—3.51 mg/kg. The content of the total 16 PAHs in the dry matter of manure was 307 μg/kg, including particular PAHs: naphthalene < 10 μg/kg, acenaphthene < 10 μg/kg, acenaphthylene < 10 μg/kg, fluorene < 10 μg/kg, phenanthrene—40 μg/kg, anthracene < 10 μg/kg, fluoranthene—59 μg/kg, pyrene—37 μg/kg, benzo(a)anthracene—25 μg/kg, chrysene—24 μg/kg, benzo(b)fluoranthene—45 μg/kg, benzo(k)fluoranthene—22 μg/kg, benzo(a)pyrene—21 μg/kg, indeno(1,2,3-cd)pyrene—18 μg/kg, dibenzo(a,h)anthracene < 10 μg/kg and benzo(g,h,i)perylene—16 μg/kg.

### Statistical analysis

The database for statistical analyses included 576 records (*n* = 576) concerning all studied years, i.e. 1998–2009. Statistical calculations were based on variance analysis with multiple measurements—three replications (blocks—B), in which two factors, namely “manure fertilisation” (O) and “diversified mineral fertilisation” (M), were regarded as constant and grouping, while the “years” (L) was treated as a factor of the multiple measurements, which was identical (confounded) with the “plant species” effect (Tables 3 and 4). Prior to the performance of statistical analyses, the normal distribution of variables within each group was verified. The homogeneity of variances in the groups was then analysed, and the equality of variance differences between measurements was verified using the *Mauchly*’s test. If necessary, multi-dimensional *Wilks*’s test and *Pillai*’s test were applied. After the performance of the *Shapiro-Wilk* test, the assumption about the normality of the analysed characteristics was rejected, which resulted in logarithmic transformation. At further stages of statistical analyses, a *post-hoc* comparison using the Tukey’s test (HSD) at *p* < 0.05 was applied. Statistical tests were conducted using STATISTICA software (StatSoft, Inc. 2014).

**Table 3:** Design of the field trial

Series with manure			Series without manure		
Block	No.	Mineral fertilisation (Table 2)	Block	No.	Mineral fertilisation (Table 2)
I	1	3	I	48	4
	2	7		47	6
	3	4		46	3
	4	1		45	5
	5	8		44	1
	6	2		43	2
	7	6		42	8
	8	5		41	7
II	9	4	II	40	5
	10	6		39	8
	11	7		38	6
	12	1		37	3
	13	8		36	7
	14	2		35	2
	15	3		34	4
	16	5		33	1
III	17	1	III	32	8
	18	7		31	5
	19	8		30	7
	20	2		29	4
	21	4		28	1
	22	6		27	5
	23	3		26	2
	24	5		25	6

**Table 4:** Two-factor variance analysis of PAH content in the design with multiple measurements

Variability source	df	16 PAHs
Manure (O)	1	**
Block (B)	2	ns
Mineral fertilisation (M)	7	**
O × M	7	**
Error 1	30	–
Years of study (L)	11	**
L × O	11	**
L × B	22	ns
L × M	77	**
L × O × M	77	**
Error 2	330	–

*ns* nonsignificant, *df* degrees of freedom

**Significance level *p* < 0.01

## Results

**Fig. 1:**
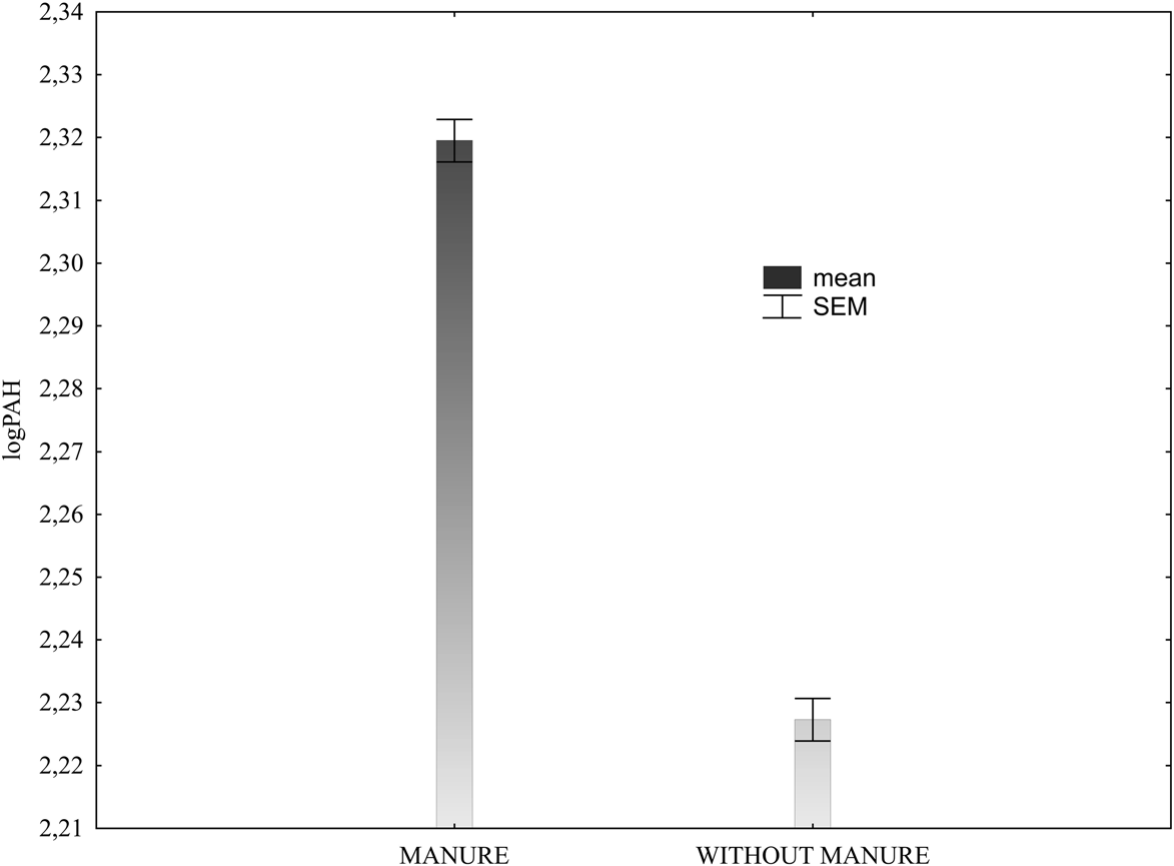
Total 16 PAH content of the soil fertilised with manure and without this fertilisation (data transformed from the years 1998–2009). SEM standard error of the mean

Generally, the content of the total 16 PAHs in soil fertilised with manure and without manure changed in similar ranges, i.e. from 28.3 to 540.8 μg/kg and 34.5 to 551.5 μg/kg, respectively (Table 5). However, maximal and mean values of the PAH content were higher in the soil with additional manure fertilisation. In such condition, the coefficients of variability (CV) in the PAH content ranged from 34.7 to 49.6%. The variability in the PAH content of the soil without manure fertilisation was higher and the CV changed in a broader range (34.6–61.9%). Statistical analyses demonstrated a highly significant effect of manure (O) and diversified mineral fertilisation (M) as well as the interaction of these factors (OxM) on the total 16 PAH content of the soil (Table 6). The content of the total 16 polycyclic aromatic hydrocarbons was significantly higher in the manure-fertilised soil than in the soil fertilised with mineral fertilisers only (Fig. 1; Table 6).

**Table 5:** Minimum and maximum content and coefficient of variability (CV) of total 16 PAH in the soil in the years 1998–2009

Objects		N_0_P_0_K_0_	N_1_P_1_K_1_	N_2_P_1_K_1_	N_3_P_1_K_1_	N_2_P_1_K_2_	N_2_P_1_K_3_	N_2_P_1_K_2_Mg	N_2_P_1_K_2_MgCa
Manure	Min (μg/kg)	43.4	28.3	124.0	87.7	77.3	96.7	42.3	87.2
	Max (μg/kg)	385.7	360.8	374.3	540.8	459.4	510.0	383.7	470.8
	CV (%)	45.2	40.4	35.5	45.9	46.4	49.6	34.7	43.4
Without manure	Min (μg/kg)	72.5	45.8	72.5	83.8	34.5	60.5	81.0	66.6
	Max (μg/kg)	318.0	331.0	341.4	338.3	362.0	551.5	321.4	287.0
	CV (%)	39.9	44.4	39.2	34.9	50.6	61.9	40.0	34.6

**Table 6:** Total 16 PAH content of the soil in the years 1998–2009 (mean for all study years) depending on manure (O), mineral fertilisation (M) and the O × M interaction

Total 16 PAH content of the soil									
Objects	N_0_P_0_K_0_	N_1_P_1_K_1_	N_2_P_1_K_1_	N_3_P_1_K_1_	N_2_P_1_K_2_	N_2_P_1_K_3_	N_2_P_1_K_2_Mg	N_2_P_1_K_2_MgCa	Mean
Manure—μg/kg	212.1	196.8	243.1	249.9	247.0	225.1	238.8	260.9	234.2
(transformed data)—log	(2.266d)	(2.237de)	(2.354ab)	(2.345ab)	(2.345ab)	(2.306c)	(2.336b)	(2.366a)	(2.320)
Without manure—μg/kg	171.9	190.3	189.9	214.9	183.3	210.1	191.3	155.9	188.4
(transformed data)—log	(2.198f)	(2.224e)	(2.241de)	(2.301c)	(2.195f)	(2.249d)	(2.244d)	(2.166g)	(2.223)
Mean—μg/kg	192.0	193.5	216.5	232.4	215.1	217.6	215.1	208.4	–
(transformed data)—log	(2.232d)	(2.231d)	(2.297b)	(2.323a)	(2.270c)	(2.277c)	(2.290bc)	(2.266c)	–

Transformed data indicated with letters differ significantly; significance level *p* < 0.05

**Fig. 2:**
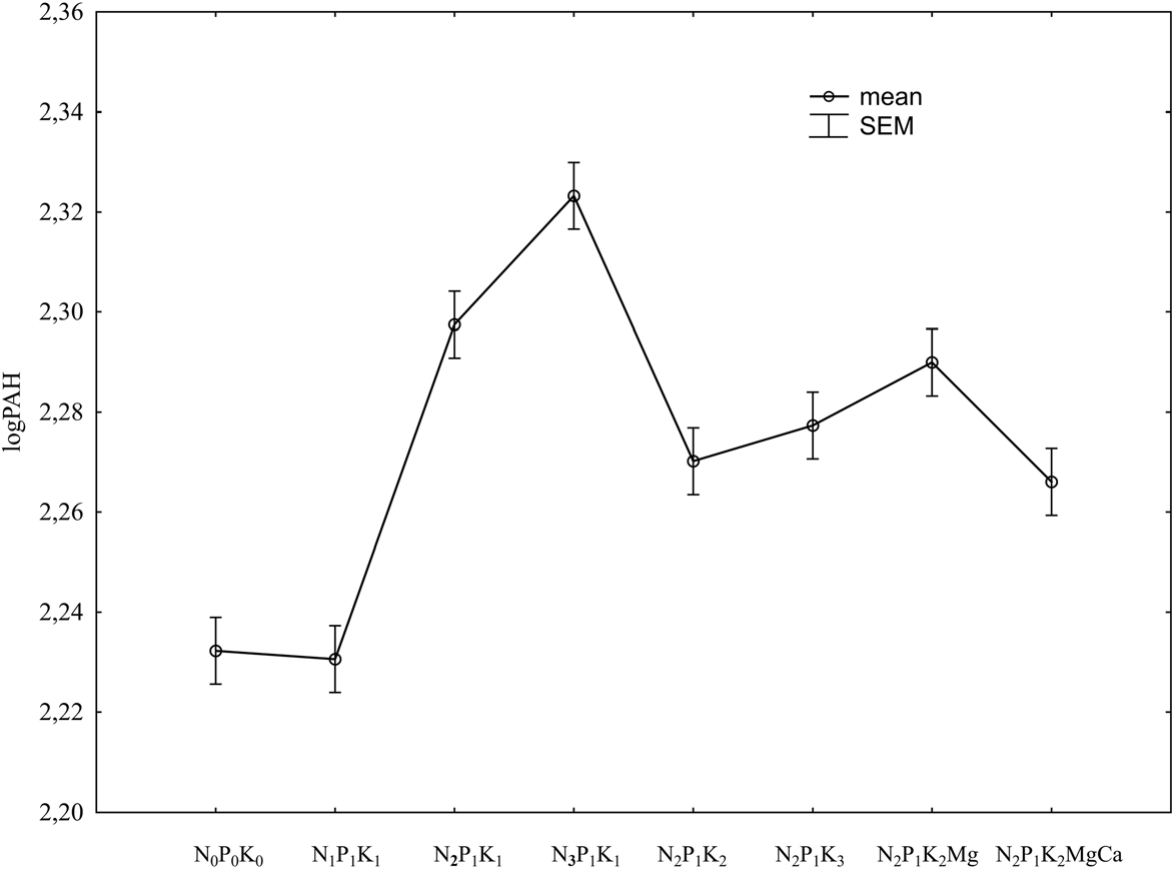
Total 16 PAH content of the soil depending on mineral fertilisation (data transformed from the years 1998–2009). SEM standard error of the mean

**Fig. 3:**
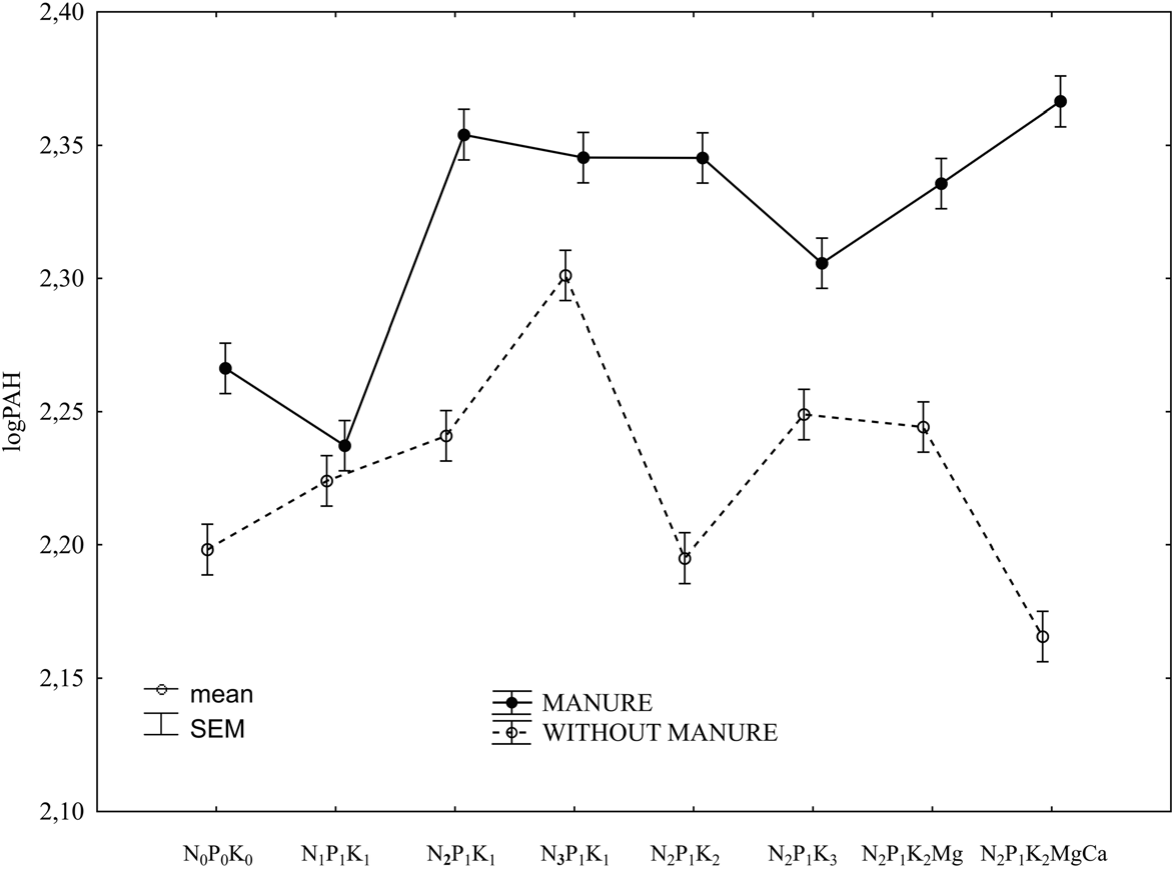
Total 16 PAH content of the soil depending on manure-mineral and mineral fertilisation (data transformed from the years 1998–2009). SEM standard error of the mean

An increase in the nitrogen dose significantly increased the total 16 PAH content of the soil with mineral fertilisation (Fig. 2; Table 6). The highest dose of nitrogen had the greatest impact. Comparing both fertilisation systems, the lowest dose of nitrogen significantly decreased its PAH content in the soil regularly fertilised with manure (Fig. 3; Table 6). The lowest nitrogen dose had a completely different effect in the soil fertilised with mineral fertilisers only, causing a significant increase in the content of the analysed substances. Nitrogen at the increased doses also resulted in an increase in the content of the analysed contaminants in both the fertilised soils, i.e. with and without manure.

The effect of potassium was not unequivocal and was found to depend on the fertilisation method, i.e. regular manure fertilisation was maintained or whether the fertilisation was carried out with mineral fertilisers only (Fig. 3). The effect of liming also depended on the fertilisation system (with or without manure). Under the regular manure fertilisation conditions, liming of the soil significantly increased the total 16 PAH content (Table 6). Lime had a completely different effect in the soil fertilised with mineral fertilisers only; a distinct decrease in the content of the analysed substances was observed, with the minimum total 16 PAH content.

## Discussion

Generally, organic fertilisers increase the organic matter content, mineral fertilisation increases the nutrient loading and improves the cation exchange capacity and liming stabilises the pH value of the environment (Włóka et al. 2015). On the other hand, fertilisers are also a potential source of contaminants, including PAHs (Włóka et al. 2014a). An increase in the organic matter content of soil promotes the accumulation of hydrophobic organic compounds, such as PAHs (Włóka et al. 2014b).

Our study confirmed a higher content of the total 16 polycyclic aromatic hydrocarbons in the manure-fertilised soil than in the soil fertilised with mineral fertilisers only. Similarly, Włóka et al. (2015) found a lower total 16 PAH content of a soil fertilised with mineral fertilisers than that of an organically fertilised soil. On the other hand, according to Ogbonna et al. (2013), manure had greater biodegradation potential for the substances in question than NPK fertilisation. The same effect of manure was reported by Agamuthu et al. (2013) and Adams et al. (2014). On the other hand, Yang et al. (2011) observed a small impact of manure on PAH degradation in the soil. The literature data presented here fail to provide an unambiguous answer to the question of manure effect on PAH content and transformations in the soil. Our research leads to the conclusion that frequent application of relatively high doses of manure (40 t/ha every two years) contributes to an increased PAH content in the soil.

Based on a multi-annual study, Mazur et al. (2004) found the highest concentration of PAHs in manure-fertilised soil, in comparison with soil fertilised with manure + PK, slurry + PK, NPK or slurry. According to Maliszewska-Kordybach (1992), the incorporation of additional organic substance to soil in the form of compost improved their stability rather than accelerate the decomposition of PAHs contained in the soil. Biodegradation of polycyclic aromatic hydrocarbons in the environment is often limited due to unfavourable nutrient conditions for bacteria. Microorganisms use polycyclic aromatic hydrocarbons as a source of carbon and energy if their amounts in the soil are insufficient (Leys et al. 2005). According to the cited authors, excess N and P may significantly limit or completely inhibit PAH decomposition in soil. Our study confirmed that an increase in the nitrogen dose significantly increased the total 16 PAH content of the soil. This effect was particularly evident after the application of maximum doses of this element in the crop rotation system. According to Acuña et al. (2012), nitrogen deficiency promotes PAH biodegradation in soil. The results do not justify a simple conclusion that a nitrogen dose may affect the PAH content of soil. It appears that nitrogen followed by regular liming of the soil had the strongest effect. Information on the positive effect of liming, such as a decrease in the content of PAHs in soil, can be found in studies conducted by Khorasanizadeh (2013), Pawar (2015) and Włóka et al. (2015).

Our study confirmed that the lowest dose of nitrogen in the soil regularly fertilised with manure was associated with a decreasing PAH content. Hence, it can be assumed that low nitrogen doses stimulate the decomposition of polycyclic aromatic hydrocarbons in soil; under such conditions, PAHs can be an attractive nutrient medium for microorganisms. However, higher concentrations of some particulate PAHs increased their toxicity to microorganisms, thereby reducing the bacterial activity (Kamil and Talib 2016). Many of the authors cited above stated explicitly that nitrogen excess had an adverse effect on the PAH decomposition in soil. However, Emami et al. (2014) demonstrated that nitrogen fertilisers could decrease the content of these compounds in the soil. On the other hand, the lowest nitrogen dose in the soil fertilised with mineral fertilisers only had the opposite effect, i.e. there was a significant increase in the content of PAHs. Under these conditions, it can be assumed that plants used the nitrogen from fertilisers very efficiently and created an environment too poor for the intensive development of microorganisms. However, the increased doses of nitrogen resulted in an increase in the PAH content in both the soil fertilised with manure and without this fertiliser. Such an increase could have been related to the pH of soil as, according to Khorasanizadeh (2013) and Pawar (2015), PAH decomposition is limited at low pH values due to the reduced count of soil microorganisms. The relevant literature also indicates that the pH and total nitrogen content have no effect on the PAH content of soil (Zhao et al. 2014). On the other hand, Włóka et al. (2014a) demonstrated a significant correlation between the PAH content and the pH of soil. According to the cited authors, this may be an effect of fertilisation, since PAHs (not substituents) are not likely to have a significant effect on the pH of soil.

The effect of potassium was not unequivocal and largely depended on whether this element was applied under the regular manure fertilisation conditions or whether the fertilisation was carried out with mineral fertilisers only. The effect of liming on the PAH content was dependent on the fertilisation system (with or without manure). Under the regular manure fertilisation conditions, soil liming significantly increased the total 16 PAH content, whereas without manure, it significantly decreased the said parameter. It can be concluded that a large supply of organic matter from manure, simultaneously providing better conditions for microbial development by liming, could result in a low degree of PAH decomposition. According to Njoku et al. (2012), manure fertilisation should be applied in moderate amounts because any excess may result in PAH-decomposing microorganisms feeding on nutrients provided with the fertiliser instead of breaking down the undesirable substances. Lime applied with mineral fertilisers only had a completely different effect. Under these conditions, when there was no organic substance excess in the soil and pH conditions were optimal, microorganisms could decompose PAHs to a greater extent. According to Wyszkowski and Ziółkowska (2009, 2013a, b), the application of lime serves an important role in mitigating the effects of soil contamination with oil derivatives.

## Conclusions

1. Regular application of high manure doses (40 t/ha every 2 years) in addition to mineral fertilisation increased the total 16 PAH content in soil, and their content was significantly higher than in soil fertilised exclusively with mineral fertilisers.
2. Higher doses of nitrogen significantly increased the PAH content in the soil fertilised additionally with manure and without it. This effect was most evident in treatments with the highest doses. On the other hand, the lowest dose of N contributed to a decrease in the PAH content in the soil with manure.
3. The impact of increasing potassium doses on the total 16 PAH content of soil was determined by the fertilisation system, i.e. there was a distinct decrease in the soil fertilised exclusively with mineral fertilisers and a slight increase in the manure-fertilised soil.
